# Characterizing the Dynamic Evolution of Interagency Collaborative Decision-Making Networks in Response to COVID-19 in China: A Policy Document Analysis

**DOI:** 10.3390/healthcare10030590

**Published:** 2022-03-21

**Authors:** Quan Cheng, Shulin Zheng, Zheng Xiong, Minwang Lin

**Affiliations:** 1 School of Economics and Management, Fuzhou University, Fuzhou 350108, China; chengquan@fzu.edu.cn (Q.C.); n190720168@fzu.edu.cn (S.Z.); 2 Department of Foreign Languages, Zhicheng College, Fuzhou University, Fuzhou 350002, China; 02113804@fdzcxy.edu.cn

**Keywords:** public health emergencies, collaborative emergency management, collaborative network, decision-making, interagency, policy document analysis, COVID-19

## Abstract

Collaborative decision-making across multiple government agencies is considered a critical and effective strategy to combat public health crisis; however, we know little about how the collaborative decision-making works and evolves during periods of crisis. To fill this lacuna, this study uncovers the structure and evolving dynamics of the network by employing a policy document analysis. Based on the policy documents, jointly issued by the agencies of Chinese central government in four phases regarding COVID-19 control, we first constructed a co-occurrence matrix of policy-issuing agencies to outline the network structure, then drew a breadth–depth matrix to identify the role evolution of agencies, and lastly built a two-mode network consisting of policy topics and agencies to determine the evolution mechanisms of policy attentions for each agency. It was found that the network structure of interagency collaboration involves three forms: discrete structure in the early phase, subgroup structure in the middle phase, and connected structure in the latter phase. Agencies embedded in the network can be categorized into three types: leading agencies, key agencies, and auxiliary agencies, with their constituent members changed as the pandemic risks are gradually becoming under control. Furthermore, each type has its own primary policy attentions, but shares some common foci in all four phases and shifts attention in the emergency management process. This study contributes to shedding light on the formation of and variations in collaborative networks in health emergencies and provides policy implications for other countries that have struggled against COVID-19.

## 1. Introduction

Public health emergencies, characterized by suddenness, publicness, harmfulness, and complexity, demand interagency cooperation, especially in the policy-making process [1,2]. The formation of emergency response policies involves multiple government agencies. Incomplete consideration is one of the obstacles to emergency response decision-making, which leads to inadequate governance measures [3]. In the context of pandemic emergencies, a holistic view is necessary for returning people’s lives to normal. To this end, policymakers should consider many aspects of post-pandemic recovery, but merely target the pandemic du jour. In a traditional hierarchical bureaucratic system, different agencies have their own separate responsibilities or missions; however, each of them is loyal to the specialized function so that they are not authorized to make emergency decisions without cooperating with other agencies [4]. However, collaborative efforts in policy-making, such as interagency information sharing and joint coordination, are inevitable due to the dynamic environment of disasters [5]. This kind of collaboration is definitely helpful to optimize health emergency responses. In practice, when multiple agencies participate in the decision-making process, they will be connected into a collaborative network which is built to achieve a common goal. During the emergency process, collaborative networks emerge and evolve [6]. Nevertheless, little remains known about the structure of collaborative emergency network, and how the network works in emergencies. Clarifying these questions will not only help to improve the understanding of collaborative emergency governance, but also provide implications for practice.

Combating COVID-19 is a suitable research sample to study the dynamic evolution of collaborative decision-making networks. It calls for a powerful government to mobilize and schedule plenty of resources, which determines that a single government agency cannot be relied on for policy-making [1]. Meanwhile, the COVID-19 pandemic has brought an unprecedented challenge to every country because no one has ever had comprehensive and advanced experience fighting such a disease. Usually, government reactions to the COVID-19 crisis are dynamic [7]. In this regard, combating COVID-19 requires policymakers to shift their attention in time to adapt to a rapidly changing pandemic situation. Thus, the effective control over outbreaks of COVID-19 hinge on adaptive government responses [8].

Combating COVID-19 in each country generally features a long-term process which allows enough time to observe how the policy-issuing agencies participate in policy-making, and how the structures of collaborative networks, constituted by multiple policy-issuing agencies, change during the emergency management process. Among these countries, China provides a good research sample to study the collaborative interagency decision-making networks for combating COVID-19. On the one hand, China was severely hit by COVID-19 but has successfully tamed the spread of virus within several months and gained plenty of experience in combating COVID-19 [9]. The efforts made by the Chinese central government, on the other hand, are praised and described as the Chinese governance model [10]. The emergency decision-making regarding COVID-19 in China is deemed as typical collaborative governance. When we pay attention to China’s policies against COVID-19, it is easy to see that most of them are issued by multiple government agencies. Furthermore, the Chinese government’s attention on emergency management was adjusted as the pandemic evolved [8], which can help us identify the dynamic evolution path of interagency decision-making networks in public health emergencies.

Emergency management remains one of the most popular fields for studying collaboration and networks [11]. Moreover, collaborative decision-making is one of the distinctive issues of collaborative emergency management [5]. Although existing studies have paid great attention to collaborative emergency governance or management, only a small number of them has focused on collaborative decision-making. Among these studies, most of them focus on the nature of decision-making in emergencies, the imperative support to decision-making system, and factors affecting the decision-making process [5,12,13,14,15]. Nevertheless, the collaborative decision-making network has received limited attention, much less its applications in health emergencies. Wu et al. (2021) first characterized collaborative emergency networks in the decision-making process based on China’s policy against COVID-19 [1]. However, this study only examined how and why the network had been formed from a static perspective. It is evident that collaborative decision-making networks are not invariable during emergencies, particularly when encountering unprecedented crises. Emergency situations are practically dynamic due to the uncontrollable nature of disasters [5]. In this regard, collaborative decision-making networks need to be adapted to uncertain crisis conditions. However, in the extant literature, questions about whether and how networks of collaborative decision-making change during public health emergency process remain unanswered.

This article probes the following questions: Did policy-issuing agencies cooperated to form different decision-making networks during the process of combating COVID-19?; How did the roles of agencies change in the dynamic network?; Additionally, how did the agencies allocate their attentions to determine the policy issues? To answer these questions, employing a policy document analysis method would be appropriate. Policy documents are the carriers of policy, which implicitly reflect policymakers’ ideologies and provide a channel to observe the policy process [16,17]. Moreover, the policy document analysis method has been widely used in research on prior public health emergencies [18,19] as well as COVID-19 [1,8]. For example, Wu et al. (2021) applied policy document analysis to characterize the patterns of China’s policies against COVID-19 [1]. Cheng et al. (2021) used policy document analysis to assess the evolution of Chinese central government attention in response to COVID-19 [8]. Therefore, the policy document analysis method can be used not only to identify the policy topics, but also to analyze the evolution of policies. Considering that policy documents published by government agencies are authoritative and can directly reflect the work dynamics of government agencies, we decided to carry out policy document analysis based on China’s policies regarding combating COVID-19 to examine the research questions. In detail, three specific studies were conducted. First, we applied a social network analysis method to show the network structure by constructing co-occurrence matrixes of policy-issuing agencies. Second, we drew a two-dimensional “breadth–depth” matrix to reveal the role changes of policy-issuing agencies. Third, we built a two-mode “agency–topic” network to unveil the policy focus of each agency and then mapped a Sankey diagram to work out the evolution mechanisms of policy topics belonging to the policy-issuing agencies.

The remainder of this article is structured as follows. Section 2 presents a review of the literature on interagency collaborative decision-making and policy document analysis in the public health emergencies. The data, methodology, and empirical analysis results are described in Section 3 and Section 4. A subsequent discussion of the results is shown in Section 5. The final section concludes with theoretical and practical implications, as well as their limitations.

## 2. Literature Review

### 2.1. Collaborative Decision-Making in Emergency

Policy capacity has become a significant indicator for measuring the performance of national-level emergency management [20,21,22]. In terms of policy capacity required by emergency management, the state’s capacity to determine what responses are chosen ranks first. In other words, better leadership and flexible decision-making are both expected in emergency management [23,24]. The dynamic environment of disasters requires multiple agencies participating together in cooperation and coordination in decision-making procedures to achieve a common goal [5]. In nature, collaborative decision-making in emergencies is thus a multifaceted phenomenon because it requires transboundary collaboration between policymakers with different expertise [25]. To define the nature of collaborative decision-making, the literature generally encompasses membership structures which range from an individual level to the organizational level [5]. The variation which can be summarized as a continuum is collaborative decision-making between politicians or policymakers at the one extreme, and at the other extreme is collaborative decision-making across different sectors. Therefore, interagency decision-making can be recognized as one type of collaborative decision-making.

In addition to focusing on the nature of collaborative decision-making, crisis management scholars also pay much attention to factors affecting collaborative decision-making process. These factors include political and institutional legitimacy [22], socio-technical structures [26], information sharing [5,26,27], communication and interaction [28,29,30], actor characteristics (i.e., number of actors, level of interdependency, level of mutual trust) [5], etc. The items mentioned above cover the environmental level, cross-sector level, and organizational level. All the influencing factors can be derived from collaborative governance theory because collaborative decision-making is the beginning of collaborative governance processes. As for the advantages, collaborative decision-making plays an increasingly significant role in improving the policy capacity of emergency response [31], and positively affect the emergency response outcome [32]. The prior disasters have made it clear that traditional crisis management characterized by hierarchy system has proved to be ineffective, especially in the policy-making process. Collaborative decision-making in emergencies brings its own advantage to the table. Moreover, the quality of collaborative decision-making has become a performance indicator to evaluate emergency response systems [33].

In terms of techniques and guidelines that provide support for decision-making in emergencies, the literature focuses on auxiliary tools for improving the analysis capacity [34], virtual reality techniques for assisting decision-making [35], collaborative mapping engines for helping with decision-making [36], knowledge management support for storing and disseminating the contextual knowledge [37], decision support tool-set for supporting multi-agency decision-making [38], socio-technical instruments for supporting social learning between different participants [39], and principles for guiding the effective collaborative decision-making [40]. These techniques and guidelines aim to connect all the parties and improve the efficiency and quality of decision-making. The literature also presents many modeling methods to work out the best collaborative decision-making strategy and forecast the consequence achieved by the decision, such as agent-based modeling [41,42] and the stochastic Petri net [43].

Although the literature on collaborative decision-making in emergencies is considerable, few studies have focused on how collaborative decision-making works. In fact, a well-coordinated network is significant for emergency decision-making. However, scholars are nearly impossible to involve in the political area; thus, inquiring the government decision-making process becomes difficult. Thus, we turn to focus on studies on emergency management networks, which can provide theoretical implications for understanding the networks of collaborative decision-making. Among these studies, two research tracks can be traced. The first one focuses on the process level and aims to explore the factors that influence the building and sustainability of collaborative decision-making networks, including interagency communication [30], information exchange [26], mutual trust, and commitment to common purpose [44]. Scholars have also devoted themselves to develop tools or methods, such as information communication technology [45,46] and task-adaptive information distribution systems [47], to ensure the networks are successful. The second one centers on the structure level, which involves network structures and their performance. In detail, some of them conduct social network analyses to identify the key actors and structural attributes in the emergency management networks [6,48,49,50,51], and some of them examine the relationships between structural attributes and network performances [50,52,53].

Regarding collaborative decision-making networks in emergencies, the relative literature is limited. As mentioned before, Wu et al. (2021) once deconstructed the network of collaborative decision-making in emergencies; unfortunately, they failed to consider the network variation in dynamic emergency situations. Investigating emergency management networks should not neglect the dynamic developments present in disaster responses. For instance, Abbasi and Kapucu (2012) applied social network analysis to investigate the evolution of inter-organizational response networks over time [54]; Robinson et al. (2013) used media reports and emergency operation plan data sources to track the evolution of collaborative emergency networks [55]. However, the unit of analysis of these studies is executive network. In this article, we take a closer look at the network of collaborative decision-making from a dynamic perspective by analyzing policy documents developed in response to public health emergencies, jointly issued by multiple government agencies.

### 2.2. Policy Document Analysis on Public Health Emergency

Policy documents, reflecting governments’ decisions, have become analytical objects increasingly used in many topics of policy research, such as policy topics evolution [56], policy change [16], policy diffusion [57], and policy evaluation [58]. On the basis of policy documents, scholars have developed a unique research method by drawing on content analysis and text analysis, which is generally called policy document analysis. Based on the adopted analytical approaches, policy document analysis can be categorized into two types: qualitative document analysis and quantitative document analysis. The former features contextual interpretations and thick descriptions, based on the researcher’s knowledge [59,60]; the latter is more prevalent in policy document research because it mainly employs bibliometric methods, which could be more objective. Two types of policy document analysis have been applied to the health research field. For instance, some scholars used qualitative document analysis to discuss the equity embedded in public health policies [61,62]. Other scholars have applied bibliometric analysis, a quantitative method widely used in literature review research, to characterize the evolution of health-related policies [63,64].

Regarding public health emergency, policy document analysis has been highly recommended due to its advantages on analyzing policy outputs and their roles play in the emergency management. Prior to the outbreak of COVID-19, scholars had applied policy document analysis to study emergency response polices of other pandemics, such as H1N1 [19] and Ebola [65]. Policy document analysis also has been used in research about national health emergency policies. For instance, Sprogis et al. (2021) analyzed the structure and processes of a rapid response system in Australia by using document analysis [66]. Regarding COVID-19, a considerable number of studies have paid attention to emergency response polices against COVID-19, and most of them employed quantitative and qualitative document analysis methods in the policy research. Some studies have focused on the evolution of COVID-19 emergency management policies during the control and prevention periods by using some topic modeling methods [1,8]. Except for quantitative analysis, researchers also have qualitatively analyzed COVID-19 control polices from the document perspective. Specifically, Yoo et al. (2020) compared the government guidelines regarding COVID-19 control across six countries and identified their major differences and similarities [67]. Benítez et al. (2020) reviewed the response policies for COVID-19 in five Latin American countries and argued that their impacts on health outcomes [68]. In addition, some studies have focused on some specific policy-making relating to the COVID-19 pandemic, such as child protection [69] and workforce governance [70].

Through reviewing the literature of policy document analysis on COVID-19, it was found that although considerable research emphasizes the importance of policy study and also conducts policy document analysis to evaluate the public health emergency policies, but few studies apply quantitative document analysis to characterize the evolution of policy-making, let alone the dynamic network structure of collaborative policy-making. Therefore, it is necessary to extend quantitative methods to clarify how government agencies collaborate in policy-making processes in public health emergencies, which can not only enrich the literature of policy document analysis, but also contribute to studies on public health emergencies.

## 3. Materials and Methods

### 3.1. Research Methods Design

**Figure 1:**
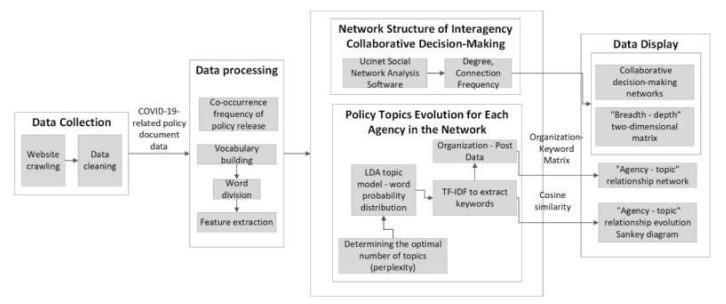
Research methods design.

This article focuses on China’s COVID-19 prevention and control practices and aims to explore the structure of the collaborative decision-making network and agency–topic evolution network. Figure 1 shows the research methods design.

### 3.2. Data Collection

This study explores the dynamic evolution mechanism of collaborative decision-making networks constructed by multiple Chinese central government agencies in response to COVID-19 (Table A1 in Appendix A shows the abbreviated name of each agency). The policy documents jointly issued by multiple Chinese government agencies can be regarded as a manifestation of collaborative decision-making. The official policy document data were extracted from the official website of the State Council of China (http://www.gov.cn/, accessed on 10 September 2020), which are available in the public domain and exempt from ethical approval. Specifically, “COVID-19” and “pandemic prevention and control” were used as the search keywords, and all the data were retrieved from 20 January 2020 to 7 August 2020. Through manual review of the crawled data, we obtained a total of 584 valid data points. The data frame included the document title, publication time, policy-issuing agency, subject classification, body content, etc.

This article focuses on the practice of COVID-19 prevention and control in China, and explores the structure of collaborative decision-making networks and agency–topic evolution networks. If we use the same time interval to divide the phases based solely on the time span, it will not be able to adequately explain the characteristics of China’s fight against the COVID-19 pandemic, which are the adopt of measures according to the time and adjusting the strategy in different phases. To effectively reveal the dynamic evolution of interagency collaborative decision-making networks, the development timeline of government efforts to combat the COVID-19 pandemic should be considered. Thus, this article refers to the White Paper “The China Action against COVID-19 Pandemic” released by the State Council Information Office of the People’s Republic of China on 7 June 2020, in which it divides the timeline of Chinese government’s efforts to combat the COVID-19 pandemic into five periods. However, the Chinese central government issued the earliest policy document concerning combating COVID-19 in the second period. Thus, we only focus on the last four periods and renamed them as “Phase T1”, “Phase T2”, “Phase T3” and “Phase T4”, respectively (Table 1).

Phase T1 preliminary features the containment of COVID-19; a milestone was the lockdown of Wuhan city. The government issued a total of 152 policy documents, but most of them were issued separately by each policy-issuing agency; only 38 policy documents were jointly issued by multiple agencies. Phase T2 features the initial control of COVID-19. A symbolic event of this stage is the number of new local COVID-19 infections dropping to single digits, and the number of documents jointly issued by multiple agencies accounted for 26.3%. Phase T3 features the overall control of COVID-19. On 18 March 2020, China reported zero new local cases for the first time, and the number of documents jointly issued by multiple agencies accounted for 34.8%. Phase T4 features regular COVID-19 prevention and control processes. On 29 April 2020, the Chinese central government made new arrangements on implementing regular COVID-19 prevention and control measures and fully advancing work resumption, and the number of documents jointly issued by multiple agencies accounted for 37.9%. In summary, the proportion of jointly issued policy documents gradually increased at each phase.

### 3.3. Methods

#### 3.3.1. Social Network Analysis

Social network analysis is a quantitative analysis method developed by sociologists based on mathematical methods and graph theory, which can be used to analyze social networks from many different aspects, such as network density analysis, centrality analysis, and core–periphery analysis [71]. The social network can intuitively display a structure composed of multiple subjects and reflect the relationships between various nodes through various indicators.

The core of social network analysis includes constructing a co-occurrence matrix of actors in the network, drawing a collaborative network, and using three centrality types—betweenness centrality, closeness centrality and eigenvector centrality—to analyze a single node. Betweenness centrality is a measure of the ability of an actor in the network to act as a network intermediary, and it reflects the degree of control of a node over resources. Closeness centrality is used to evaluate the closeness between one node and other nodes in the network. Eigenvector centrality is used to determine the core nodes of the network. The basic idea is that the centrality of a node is a function of the centrality of adjacent nodes. It is believed that the importance of a node depends on the number of neighbors and the importance of neighboring nodes.

#### 3.3.2. Text Mining

Text mining is based on computational linguistics and statistical mathematical analysis, combined with machine learning and information retrieval technology, to discover and extract hidden knowledge from text data in a document set independent of user information needs. It is a process of text information description to selection and extraction modes, and finally the formation of user-understandable information knowledge.

We employed the LDA (latent Dirichlet allocation) topic-training model to extract the topics of the policy documents to explore the evolution of their foci. LDA is a document topic generation model proposed by David M. Blei [72]. In essence, it is based on the three-layer Bayesian probability model of “document–topic–word” and the algorithm adopting the bag-of-words model method. Each document is regarded as a word frequency vector; each document represents the probability distribution of some topics; and each topic is the probability distribution of many words.

We selected TF-IDF (term frequency–inverse document frequency) to calculate feature weights. TF-IDF is a method of calculating feature weights in text classification. TF stands for word frequency, which is used to count the frequency of each word in the text and reflect the ability of the word to describe the content of the document. IDF is called the inverse document probability, which is used to evaluate the universality of each word to the corpus.

Selecting an appropriate number of topics has a greater impact on topic extraction and topic strength. For the LDA model, the two most commonly used evaluation methods are Perplexity and Corre. Perplexity (PP) is used to evaluate the quality of a language model. Perplexity can be understood as how uncertain the trained model is about which topic the document d belongs to for an article d. This uncertainty is the perplexity. The lower the perplexity, the better the clustering effect. For models trained on different topics, its perplexity is calculated. The topic corresponding to the minimum perplexity is the optimal number of topics. This study chose perplexity as the basis for determining the optimal number of topics. The formula for calculating *perplexity* is as follows:

(1) $$Perplexity=exp\left(-\frac{\sum_{d=1}^{M}{log}_{2}P\left(w_{d}\right)}{\sum_{d=1}^{M}N_{d}}\right)$$

where *M* is the number of documents, *N_d_* is the set of entries in each document, and *P(w_d_)* is the probability of generating each entry.

#### 3.3.3. Sankey Diagram

A Sankey diagram, also known as a Sankey energy split diagram or a Sankey energy balance diagram, is a specific type of flow chart that shows the use of thermal energy and the flow of energy in a very vivid way. Sankey diagrams were originally used in energy management and the chemical industry. With the development of society, they have also been widely used in some emerging industries and financial institutions. The complex data can be displayed more clearly through the use of a Sankey diagram, and the connection strength relationship between the co-words of different keywords at different stages can be more clearly displayed, the time series information of the data can be more effectively conveyed, and the potential information can be mined and visualized.

This article refers to the time series text visualization method based on a Sankey diagram. It adopts the probability distribution of the topic-word in the LDA to calculate the similarity between the topic-words and reveals the topic evolution in different stages. First, we determined all the high-frequency feature terms and selected the representative terms of each phase, and then converted them into bow vectors. Second, the bow vectors were converted through the TF-IDF model. Finally, a cosine similarity was used to calculate the topic similarity among adjacent phases. The calculation formula is as follows, where two attribute vectors *A* and *B* are given, attribute vectors *A* and *B* are usually word frequency vectors in the document, and *A_i_* and *B_i_* represent the components of the vectors *A* and *B*, respectively.

(2) $$Similarity=\mathrm{Cos}\left(\theta \right)=\frac{A\cdot B}{\left||A||\;||B|\right|}=\frac{\sum_{i=1}^{n}A_{i}\times B_{i}}{\sqrt{\sum_{i=1}^{n}{\left(A_{i}\right)}^{2}}\times \sqrt{\sum_{i=1}^{n}{\left(B_{i}\right)}^{2}}}\;$$

## 4. Results

### 4.1. Network Structure of Interagency Collaborative Decision-Making

**Figure 2:**
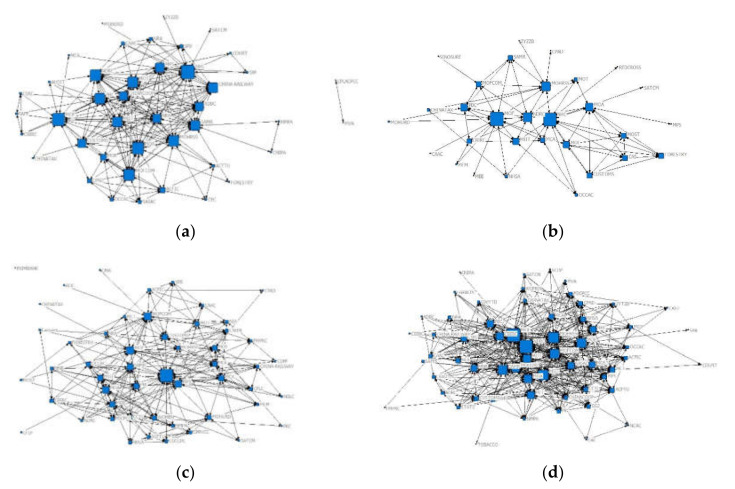
Collaborative decision-making networks in each phase. (**a**) T1 (20 January 2020–20 February 2020); (**b**) T2 (21 February 2020–17 March 2020); (**c**) T3 (18 March 2020–28 April 2020); (**d**) T4 (29 April 2020–7 August 2020).

The number of policy-issuing agencies involved in each phase can change, and the type of collaborative decision-making networks can vary in different phases of emergency governance [73,74]. To explore the structural variation of collaborative decision-making networks, a social network analysis is employed. Based on the data of the policy-issuing agencies involved in each phase, this study constructed co-occurrence matrices of policy-issuing agencies to outline the network structures and then used social network analysis software to draw the collaborative decision-making networks in each phase. The result is shown in Figure 2. Among them, the blue squares represent policy-issuing institutions, the size of the squares represents the number of times the institution has issued policies, and the lines between the squares indicate that there is a joint policy-issuing relationship between institutions. The network densities of collaborative decision-making in the four phases were 0.3043, 0.1954, 0.2323, and 0.5164, which indicated that the interagency collaborative decision-making network is dynamically changing in the emergency management process.

In the first phase, the core agencies involved are relatively fixed, and they are connected to peripheral agencies to form a relatively stable but discrete network structure. The National Health Commission (NHC), Ministry of Finance (MOF), Ministry of Transport (MOT), Ministry of Commerce (MOC) and Ministry of Human Resources and Social Security (MOHRSS) are clustered in the center of the network, and the distance between the agencies is relatively small. The MOF and National Development and Reform Commission (NDRC) have high betweenness centrality (274.129) and high closeness centrality (29.927 and 29.496, respectively) and have a high eigenvector centrality (0.356), which indicates that these two agencies play a “bridge” role, they have high degrees of independence, and their collaborative partners are relatively important.

In the second phase, the collaborative decision-making network formed two major subnetwork structures. NHC is connected to the Ministry of Education (MOE), Ministry of Industry and Information Technology (MIIT), Ministry of Science and Technology (MOST), Ministry of Transport (MOT), and MOHRSS. MOF is connected to NDRC, MIIT, People’s Bank of China (PBC), China Banking and Insurance Regulatory Commission (CBIRC), MOC, National Taxation Bureau (NTB), etc. NHC and MOF are 133.56 and 119.917, which are significantly higher values than those of other nodes, and function as the “bridge”. In addition, the indicators of MOF (i.e., degree centrality: 19, closeness centrality: 64.043, betweenness centrality: 133.56, eigenvector centrality: 0.45) are higher than those of NHC (i.e., degree: 15, closeness centrality: 59.184, betweenness centrality: 119.917, eigenvector centrality: 0.239), which indicates that MOF played a more significant role in collaborative decision-making in the second phase.

In the third phase, a collaborative network structure with NHC as the core was formed, and MOHRSS, MOC, MOT, MOF, and the State Administration for Market Regulation (SAMR) ranked in level 2. In level 3, they linked to agencies with similar functions. For example, MOT is linked to the Ministry of Transport (MOT) and General Administration of Customs (CUSTOMS), Civil Aviation Administration (CAAC), State Post Bureau (SPB), and the Ministry of Foreign Affairs (FMPRC). The NHC node in the network has the highest indicator values, with a degree centrality of 61 and a betweenness centrality of 346.104, which are remarkably distinguished from other policy-issuing agencies. In this phase, the NHC functioned as a leader in coordinating affairs and coordinated with other agencies to promote work arrangements combating COVID-19.

In the fourth phase, the scale of the involved agencies was further expanded; the network density reached the highest; the involved agencies were diversified; and the collaboration between agencies was in a balanced state. The collaborative decision-making network formed a connected structure. NDRC, MOF, MOHRSS, and MITT occupy the important nodes of the network. Their values of closeness centrality, betweenness centrality, and eigenvector centrality are relatively close.

Overall, as COVID-19 pandemic emergency management developed, the number of policy-issuing agencies expanded from 44 in the first stage to 59 in the last stage, and the links between the agencies became closer and more stable.

### 4.2. Role Evolution of Agency in the Collaborative Decision-Making Network

**Figure 3:**
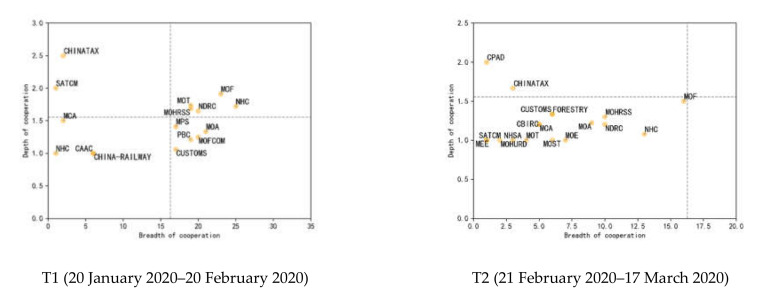

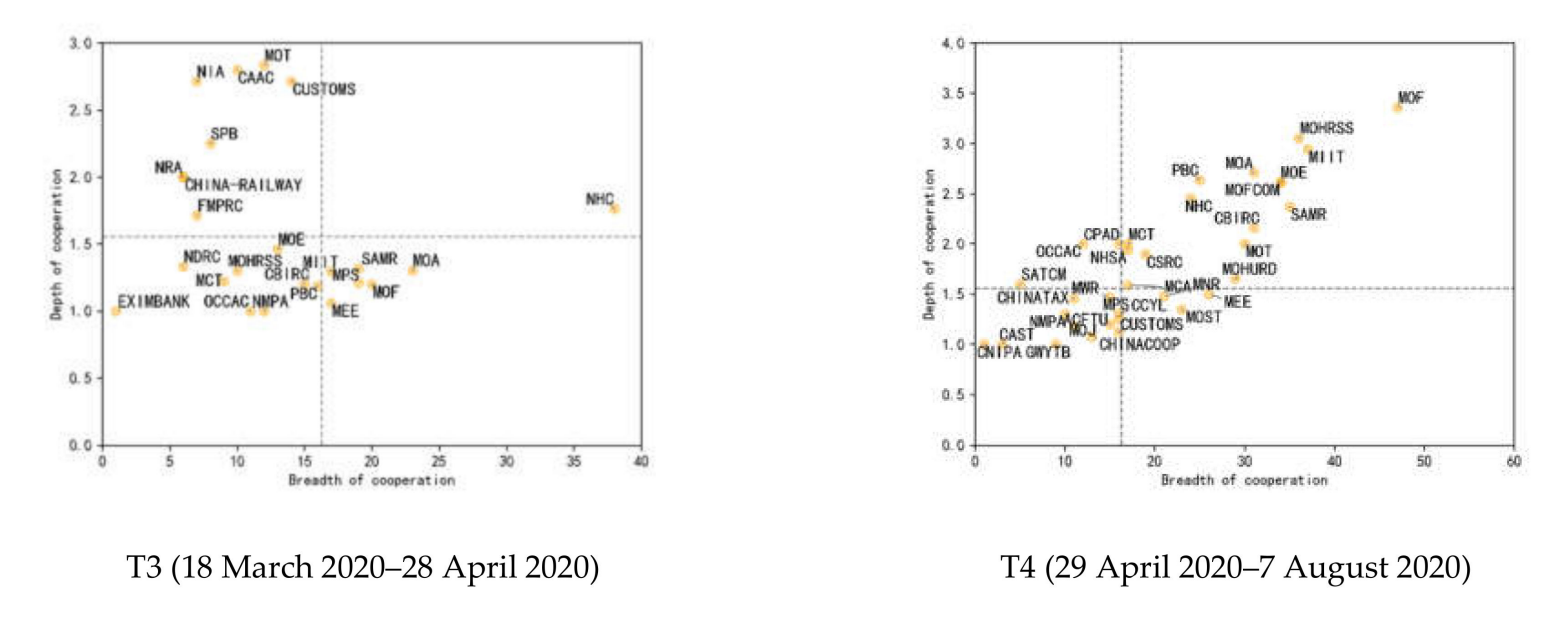
Two-dimensional “breadth–depth” matrix.

On the basis of the above research results, it is found that NHC, MOF, NDRC, MOT and MOHRSS are core participants in the collaborative decision-making network. To further explore the changing role of each agency, this study drew two-dimensional “breadth–depth” matrices based on the degree of nodes and the frequency of links. The degree of the node indicates the number of partners being connected to one single policy-issuing agency, which can be used to measure the “breadth” of collaboration. The higher the degree, the wider the range of collaboration, and the bigger the role the agency played. The frequency of links indicates the frequency of an agency collaborating with other agencies in decision-making. Considering that the frequency of links is affected by the degree of nodes, this study uses the ratio of the frequency of links to the degree of nodes to measure the “depth” of collaboration. Then, four two-dimensional “breadth–depth” matrices in four phases are constructed. The results are shown in Figure 3.

Figure 3 presents the collaborative networks of policy-issuing agencies in each phase. In a two-dimensional matrix, the subjects in the first quadrant and far away from the mean lines are regarded as leading subjects who play the leading role in a collaborative network. As defined, the leading subjects are the agencies who not only collaborated with many other agencies in policy-making, but also participated in collaborations many times. The subjects located in other quadrants but close to the mean lines are regarded as key subjects. As defined, the key subjects are the agencies who either collaborated with many other agencies or participated in collaborations many times. The subjects located in other quadrants and close to the horizontal or vertical axis are regarded as auxiliary subjects. As defined, the auxiliary subjects are agencies who have collaborated with fewer agencies in the decision-making although are rarely also involved in the collaborations. Table 2 shows the evolution of the roles of policy-issuing agencies in the collaborative decision-making network at four stages. Although the structural differences of each agency in the collaborative networks have been identified, we still cannot judge their level of influence. Borrowing the idea from the study made by Wu et al. (2021) [1], we further analyzed the administrative influence agencies. The leading nodes in the collaborative networks have the strongest influence on the other agencies. The key nodes in the networks have a strong influence on the other agencies. The auxiliary nodes have a weak influence on other agencies. Thus, according to the administrative influence of agencies involved in policy-making in response to COVID-19, the agencies can be categorized into three types—in descending order: leading agencies, key agencies, and auxiliary agencies.

Evidently, in the early phase, the breadth and depth of collaborative decision-making networks were relatively low; the scale and scope of cooperative co-governance were relatively small; the strength of collaboration among the agencies was weak; the extensibility was insufficient; and the structure of the collaborative network was relatively unstable. With the strengthening of COVID-19 prevention and control efforts, the number of policy-issuing agencies gradually increased, as well as the breadth and depth of the collaborative decision-making networks. Some agencies evolved into key agencies in the networks. It is worth noting that the leading agencies in the second and third phases were single, and their networks featured multiple agencies led by only one leading agency. In addition, in the early phase, the number of key structural agencies was higher than the number of key functional agencies. In the later phase, however, the number of key functional agencies was on the rise. The form of collaboration gradually evolved from a symbolic style to a complementary style. Collaborative emergency governance had emerged. In the last phase, the number of leading agencies increased significantly, and a clear distinction existed among leading agencies, key agencies, and auxiliary agencies.

### 4.3. Policy Topics Evolution for Each Agency in the Network

The aforementioned single-mode collaborative decision-making network revealed the structural characteristics. However, to obtain knowledge about how agencies participate in different policy topics during different phases of the COVID-19 pandemic emergency management, an “agency–topic” two-mode network needed to be constructed. In detail, this study first drew the document–term matrices by calculating TF-IDF based on 584 jointly issued policy documents as source data and linked agencies to high-frequency feature words, which could be generalized into specific topics. If a co-occurrence existed between policy-issuing agency O and term T, it was recorded as “1”; otherwise, it was recorded as “0”. Then, we constructed the agency–term co-occurrence matrices and draw the two-mode “agency–topic” network using Gephi, which is complex network analysis software. In the networks, green nodes represented agencies and red nodes represented topics.

#### 4.3.1. “Agency–Topic” Relationship Network Construction in the First Phase

**Figure 4:**
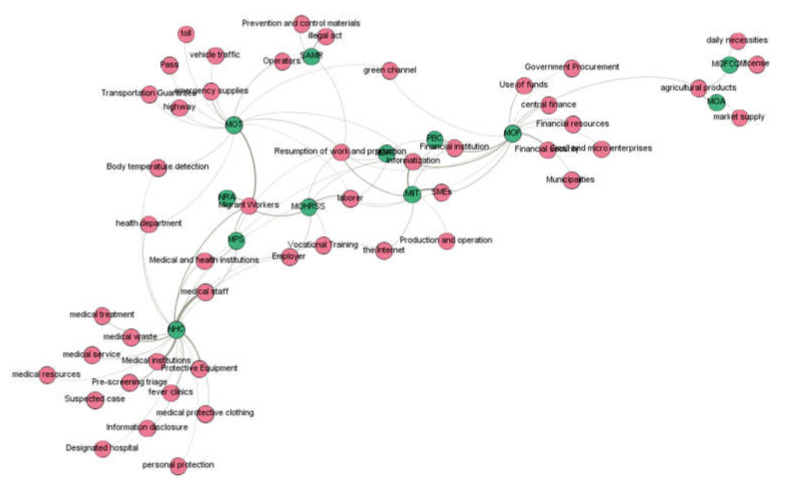
“Agency–topic” relationship network in the first phase.

Figure 4 shows the topic relationship of the COVID-19 policies jointly issued by the MOF, NHC, and other agencies in the first phase. In the networks, green nodes represented agencies and red nodes represented topics. From the connected keywords, we could determine the work themes. From Figure 4, we found that the work themes at this stage are mainly related to the emergency prevention and control of the epidemic, and the work themes of each institution vary according to their functions, as shown in Table 3.

#### 4.3.2. “Agency–Topic” Relationship Network Construction in the Second Phase

**Figure 5:**
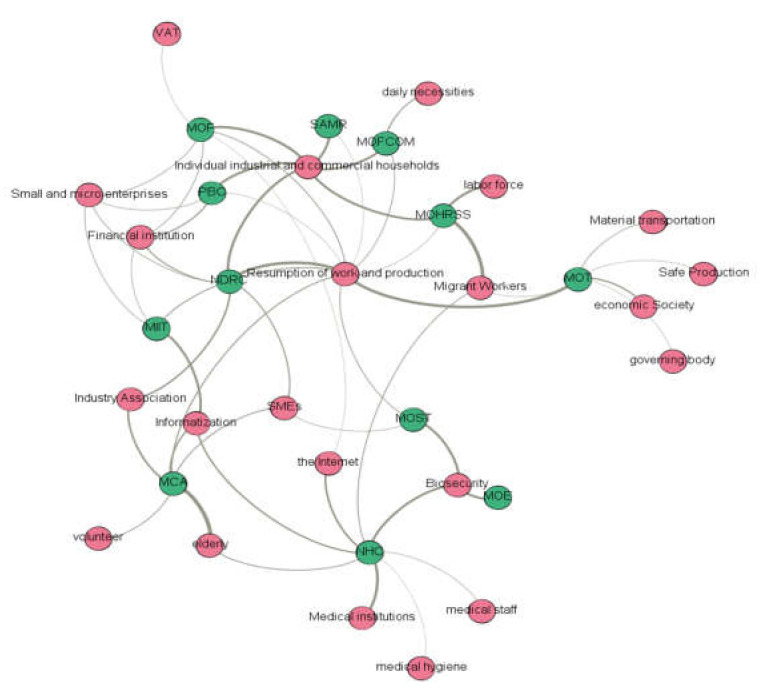
“Agency–topic” relationship network in the second phase.

Figure 5 shows the topic relationship of the policies combating COVID-19 jointly is-sued by the MOF, NHC and other core agencies in the second phase. The policy topics are mainly related to the resumption of work and production. Table 4 displays the results of Figure 5 in table format. In the networks, green nodes represented agencies and red nodes represented topics.

#### 4.3.3. “Agency–Topic” Relationship Network Construction in the Third Phase

**Figure 6:**
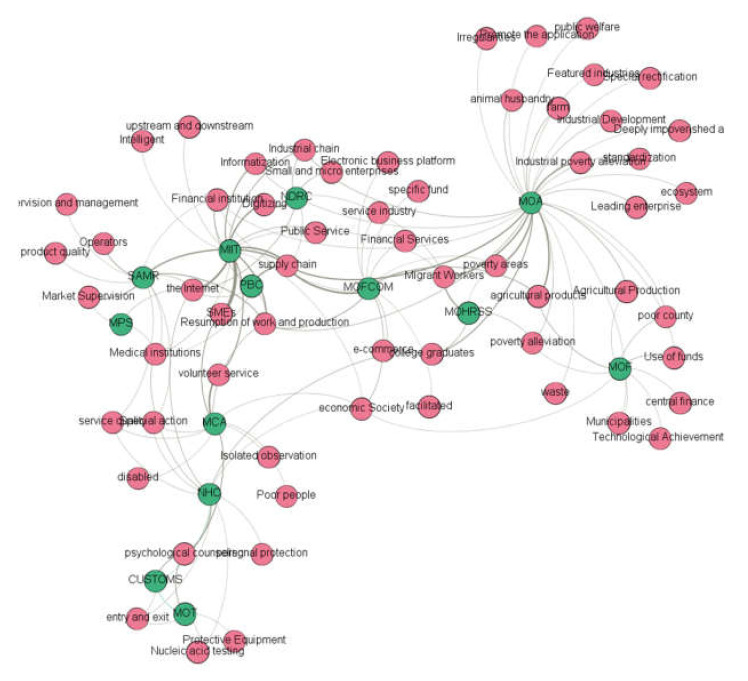
“Agency–topic” relationship network in the third phase.

Figure 6 shows the topic relationship of the policies combating COVID-19 jointly is-sued by the MOF, MOA, and other core agencies in the third phase. This stage shows three major work themes, namely, industrial poverty alleviation and poverty alleviation, digital construction and informatization development, and local epidemic prevention and control management. In the networks, green nodes represented agencies and red nodes represented topics. The themes of work of each agency are shown in Table 5.

#### 4.3.4. “Agency–Topic” Relationship Network Construction in the Fourth Phase

**Figure 7:**
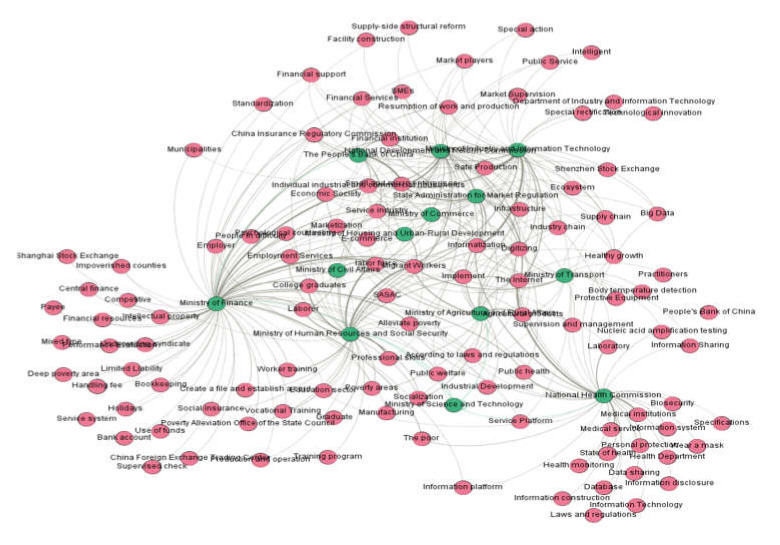
Agency–topic relationship network in the fourth phase.

Figure 7 shows the topic relationship of the policies combating COVID-19 jointly issued by the NHC, MST, and other core agencies in the third phase. Table 6 displays the result of Figure 7 in table format. In the fourth phase, COVID-19 pandemic prevention and control in China had become regular, and government attention had changed to protecting the achievements and recovering economic and social development. It is a remarkable fact that information technology has caught the policy attention of government agencies in this phase. In the networks, green nodes represented agencies and red nodes represented topics. The agencies and their work foci are detailed in Table 6.

#### 4.3.5. “Agency–Topic” Relationship Evolution

Based on the topic–term probability distribution in the LDA, we continued to calculate the similarity between topic terms to measure the topic correlation among adjacent phases and reveal the evolution of topic foci across different phases.

**Figure 8:**
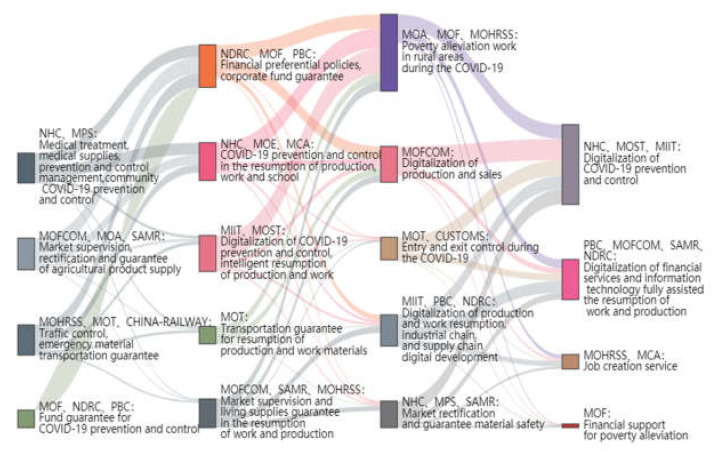
“Agency–topic” relationship evolution Sankey diagram.

The topics are summarized according to topic similarity and high-frequency words. On this basis, a Sankey diagram is drawn to achieve visualization because it is a much more readable way to show the topic evolution in each phase (see Figure 8). The Sankey diagram is mainly composed of edges, flows and fulcrums, where the edges represent the flowing data, the flows represent the specific values of the flowing data, and the nodes represent different classifications. The column in Figure 8 indicates the various phases of combating COVID-19; the element blocks in the same column represent the concerning topics in the phase; and the size of the element blocks indicates the degree of similarity between the topic in the current phase and the topics in the next phase, and the rectangular blocks with different colors represent the flow paths of the government agencies’ work topics.

From observing the whole period of combating COVID-19, NHC and MOF could both clearly be seen in every phase of collaborative emergency decision-making. The policy topic foci of NHCs evolved along the lines of “medical treatment and prevention and control management → pandemic prevention and control during resumption of production and work → regular pandemic prevention and control → digitization of pandemic prevention and control”, and their cooperation agencies adjusted with the change in policy focus. In the early phase, the NHC collaborated with the MPS to arrange medical treatment and prevention and control management. Subsequently, the NHC worked with the MOE and MOHRSS to clarify the requirements for pandemic prevention and control in the resumption process of work and production. As combating COVID-19 achieved staged success, NHCs joined forces with SAMR and MPS to regulate market disorder. When COVID-19 prevention and response became routine, the NHC worked with MOIIT and MST to promote the digitalization of pandemic prevention and control.

The policy attention of the MOF, NDRC, and PBC was relatively fixed and mainly focuses on financial supports and guarantees. With the improvements in epidemic prevention and control situations, they have gradually realized the transition from emergency prevention and control to promoting the economic recovery of enterprises and then to the normalization of maintaining stability. In the end, two evolutionary paths were formed. The first path was “Funds guarantee for epidemic prevention and control → Financial preferential policies, enterprise fund guarantee → Poverty alleviation during the epidemic period → Financial support for poverty alleviation”, and the second path was “Funding guarantee for pandemic prevention and control → Financial preferential policies, corporate fund guarantee → Digitization of resumption of production and resumption, digitization of industrial chain and supply chain → Financial service digitization, information technology assisting the resumption of work and production”.

It is worth noting that although MIIT and MOST did not appear in the first stage, since the second stage, they have set out to promote the integration of information technology into various tasks. Policy foci, such as the digitalization of pandemic prevention and control and the intelligentization of the resumption of work and production, began to appear and then evolved into other policy foci, such as the digitalization of production and sale, the digitalization of the resumption of work and production, and the digital development of industries and the supply chain. In the final phase, the policy focuses converged on the digitization of epidemic prevention and control. Specifically, the digitization of financial services and integration information technology into the resumption of work and production became two major tasks in the later phase.

## 5. Discussion

### 5.1. Agency Role, Policy Issues, and Decision Network in Combating COVID-19

Through social network analysis and topic evolution path based on the emergency policies against COVID-19 jointly issued by Chinese government agencies, we not only classified the structure of collaborative interagency decision-making networks in combating COVID-19, but also found that an evolution path existed in the dynamic network. The Chinese experience of combating COVID-19 can be summarized into many features, such as building an adaptive public health emergency system [8]. In contrast to existing studies, this study attempted to summarize the Chinese experience of combating COVID-19 from a new perspective, namely, collaborative emergency management. For China itself, the experience of drawing from the government’s efforts against COVID-19 is beneficial to emergency public health management in the future. For other countries suffering as a result of COVID-19, the Chinese experience could also provide several valuable and feasible tactics to contain pandemics, and help with more rapid recovery.

First, the government should pay different attention to different issues according to changing environments. As we know, government attention is a limited resource when coping with public health crises [8]. This means that only some important issues will be addressed in the policy-making agenda. Moreover, the dynamic evolution of public health crises determines that governments would suffer from different problems in different phases. It requires the government to pool limited resources to solve urgent problems within a specific time period. Take combating COVID-19 in China as an example: medical treatment was the top priority in the early phases, which required health-related agencies to play the leading role. Instead, economic recovery became the primary task in the later phases, and economic-related agencies took over leading responsibilities. In summary, emergency public health management needs multiple government agencies to serve in different roles to come up with specific policy implementation plans.

Second, the government should balance the attention given to COVID-19 control and economic recovery. In practice, some countries attached too much importance to the prevention and control of COVID-19, resulting in a serious impact on economic development. In contrast, some countries put too much emphasis on economic recovery while ignoring the prevention and control of COVID-19, which resulted in substantial increases in the COVID-19 infection rate and death rate. The experience in China showed that we should balance the attention given to COVID-19 control and economic recovery. Once new local cases are found to spread, the government should respond quickly and implement stricter prevention and control measures, such as quarantines and lockdowns. As long as the spread is under control, the government should set out to return people’s lives to normal and promote the resumption of work and production.

Third, a connected collaborative decision-making network is needed in public health emergencies. Collaboration is the basic requirement for emergency decision-making. Government agencies should cooperate in a close network. As we know, different agencies have different responsibilities and professional abilities. Although collaborative interagency decision-making networks exist in many countries suffering from the COVID-19 pandemic, their structures are always somewhat loose. As for China, the emergency collaborative decision-making network was also discrete in structure in the early phases; however, the network structure evolved into a connected form in the later phases, which meant there were many government agencies acting as key moderators to connect all the policy-issuing agencies together. In other words, it not only requires multiple actors to participate in the decision-making process in public health emergencies, but also needs some government agencies to shoulder coordination responsibilities.

Finally, shared leadership at the national level needs to be gradually switched and expanded during crisis management. From China’s experience of combating COVID-19, the collaborative interagency decision-making network was switched to adapt to the changing situation, which makes us reflect on the leadership in crisis management. Evidently, adaptive leadership is needed in combating public health crises. Take the COVID-19 pandemic as an example: it has brought about many wicked challenges. Some of them have never happened before, and require a rapid and comprehensive government response. Nevertheless, the emergency leadership in most countries would invariable so that they would suffer from a lack of flexibility. In contrast, the emergency decision-making network in China exhibits adaptability, which contributes to building leadership competency. There is an argument that leadership competency has a positive relationship with the effectiveness of crisis management [75]. China’s experience shows that adaptive leadership in crisis management plays a crucial role in combating COVID-19, and significantly affects the effectiveness of emergency plans.

### 5.2. Limitations

The present study has several limitations. First, the research sample of this study was limited to the Chinese central government level. The response to major public health emergencies not only depends on the horizontal collaborative decision-making of central agencies, but also depends on the vertical collaborative work of central and local agencies at all levels. This study ignored the collaborative relationship between local governments and the central government in coordinating the COVID-19 response.

Second, although policy document analysis is an effective method to identify agency collaboration, the sudden characteristics of COVID-19 have determined the real-time and dynamic nature of collaborative decision-making across agencies. It only takes the joint documents published by the central government regularly as objects of analysis; thus, it will ignore the real-time characteristics of multi-sectoral collaborative decision-making in the process of responding to COVID-19. At the same time, other important factors in the collaborative response of various agencies will also be ignored.

Third, in this study, the LDA model was used to extract the topics of policy texts jointly issued by governments, whereas more powerful semantic mining models, such as Word2Vec and LDA2evc, have not been applied. On the one hand, the effect of topic mining needs to be improved; on the other hand, due to the limitations of the LDA clustering algorithm, the topic extraction of policy text has strong subjectivity.

In follow-up studies, collaborations across different levels and different factors are also notable for inquiry. As for research methods, subsequent studies could consider employing other text mining techniques, such as Word2Vec and LDA2evc.

## 6. Conclusions

Professionalism and adaptive governance capacity are embodied in the Chinese governmental effort to combat COVID-19. Chinese emergency public health management features unified commands, adaptive responses, and collaborative governance. China’s experience helps to shed light on the necessity of interagency collaborative governance in public health emergencies. It is crucial to introduce the collaborative governance concept to the policy-making process, as well as in the policy implementation process. Collaborative governance in emergency management requires government agencies to clarify their own responsibilities and labor division to formulate an effective joint prevention and control mechanism. In responding to the COVID-19 pandemic, Chinese government agencies quickly integrated resources and established collaborative interagency decision-making networks that varied throughout different phases of emergency pandemic management.

This study explored the dynamic interagency collaborative decision-making network, characterized structural variation and agency role change, and identified the evolution of policy topics for each agency through policy document analysis. The results show that there are three forms of network structure in the emergency process: discrete structure in the early phase, subgroup structure in the middle phase, and connected structure in the later phase. Agencies embedded in the network can be categorized into three types: leading agencies, key agencies, and auxiliary agencies. Furthermore, agencies each have their own primary policy focus, but share some common foci across all four phases and shift their attention in the emergency management process. These research findings can provide implications for understanding collaborative decision-making in public health emergencies by summarizing the Chinese experience of combating COVID-19. Understanding the dynamic evolution of collaborative decision-making networks can also help to obtain knowledge about how to improve emergency management ability when facing public health emergencies.

## Figures and Tables

**Table 1:** Four phases of managing the COVID-19 pandemic in China.

Phase	Time Span	Phase Feature	Number of Documents	Separately Issued	Jointly Issued
T1	20 January 2020–20 February 2020	Preliminary containment	152	75%	25%
T2	21 February 2020–17 March 2020	Initial control	118	73.7%	26.3%
T3	18 March 2020–28 April 2020	Overall control	132	65.2%	34.8%
T4	29 April 2020–7 August 2020	Regular prevention and control	182	62.1%	37.9%

**Table 2:** Role evolution of agencies involved in the network.

Phase No.	Leading Agencies	Key Agencies	Auxiliary Agencies
T1	MOF, NHC, NDRC, MOT, MOHRSS	CAAC, MOFCOM, MPS, PBC, CHINA-RAILWAY, CUSTOMS, MOA	CHINATAX, SATCM, MCA, CHINACOOP
T2	MOF	CBIRC, MCA, NHC, MOHRSS, NDRC, CUSTOMS, MOA, FORESTRY	CPAD, CHINATAX, SATCM, MEE, MOHURD, MOT, NHSA, MOST, MOE
T3	NHC	MOT, CUSTOMS, CAAC, NIA, SPB, NRA, CHINA-RAILWAY, FMPRC, MOE, MOF, MIIT, SAMR, MOA, CBIRC	NDRC, MOHRSS, MPS, PBC, OCCAC, MEE, NMPA, EXIMBANK, MCT
T4	MOF, NDRS, MOHRSS, MIIT, MOA, NHC, MOFCOM, MOE, CBIRC, SAMR, MOT, PBC	CSRC, MCT, CPAD, NHSA, MCA, MOHURD, MWR	OCCAC, SATCM, CHINATAX, NMPA, CNIPA, CAST, CUSTOMS, MNR, MEE, MOST, ACFTU, CCYL, CHINACOOP, GWYTB, MOJ, MPS

**Table 3:** Policy topics of interagency collaborative decision-making in the first phase.

Involving Agencies	High-Frequency Feature Words	Policy Topics
NHC and MPS	Medical institution, medical service, fever clinic, personal protective equipment, pre-examination, and triage	Medical treatment and prevention and control
MOF, NDRC, and PBC	Use of funds, fund guarantee, government procurement	Fund guarantee for epidemic prevention and control
MOC, MOA, and SAMR	Illegal behavior, pandemic prevention materials, license, and market supply and	Market supervision and guarantee of agricultural product supply
MOHRSS, MOT, CHINA-RAILWAY	Resumption of work and production, students’ resumption of classes, personnel returning to work, body temperature detection, highway, emergency supplies, transportation guarantee	Traffic control and emergency material transportation guarantee

**Table 4:** Policy topics of interagency collaborative decision-making in the second phase.

Involving Agencies	High-Frequency Feature Words	Policy Topics
NDRC, MOF, and PBC	Small and micro-enterprises, financial institutions, resumption of production and work	Financial incentive policies
NHC, MOE, and MCA	Medical and health care, medical staff, medical institutions, volunteers, senior citizens, biosafety	Epidemic prevention and control in the resumption of production, work and school
MIIT and MOST	Informatization, Internet	Digitalization of epidemic prevention and control
MOT	Material transportation, safe production	transportation guarantee for resumption of production and work materials
MOC, SAMR, and MOHRSS	Individual industrial and commercial households, daily necessities, labor, migrant workers, resumption of work and production	Market supervision in the resumption of work and production

**Table 5:** Policy topics of interagency collaborative decision-making in the third phase.

Involving Agencies	High-Frequency Feature Words	Policy Topics
MOA, MOF, and MOHRSS	Central finance, poverty-stricken counties, poverty alleviation, agricultural production industry poverty alleviation, industrial development, deeply impoverished areas, promotion and application, characteristic industries, migrant workers	Industrial targeted poverty alleviation in rural areas during the pandemic
MOC	e-commerce, e-commerce platform, facilitation, service industry, economic society	Digitalization of production and sales
MOIIT, PBC, and NDRC	Digitization, informatization, resumption of production and work, supply chain, industrial chain, small and medium-sized enterprises	Digital application of pro-duction and supply chain
NHC, MPS, and SAMR	Product quality, supervision and management, service quality, medical institutions, operators	Market regulation and guarantee material safety
MOT and CUSTOMS	Entry–exit, nucleic acid test, protective equipment	Entry and exit control during the epidemic

**Table 6:** Policy topics of interagency collaborative decision-making in the fourth phase.

Involving Agencies	High-Frequency Feature Words	Policy Topics
NHC, MOST, and MOIIT	Technical specifications, information technology, data sharing, information disclosure, informatization construction, information systems, databases, public health, information platforms	Digitalization of epidemic prevention and control
PBC, MOC, SAMR, and NDRC	Small and micro enterprises, financial institutions, financial services, financial support, safe production, resumption of work and production, small and medium-sized enterprises, informatization, Internet, digitalization	Digitalization and information technology applied to the resumption of work and production
MOHRSS and MCA	Laborer, college graduates, employment services, vocational skills, training plans, vocational training for employers	Job creation service
MOF	Poor counties, deeply impoverished areas, central finance, service system	Financial support for poverty alleviation

## Data Availability

The data presented in this study are available on request from the corresponding author. The data are not publicly available due to privacy restrictions.

## Appendix A

**Table A1:** The abbreviations of Chinese central government agencies.

Ministry	Abbreviation	Ministry	Abbreviation
Ministry of Finance	MOF	Office of Poverty Alleviation	CPAD
Ministry of Industry and Information Technology	MIIT	Taiwan Affairs Office of the State Council	GWYTB
Ministry of Public Security	MPS	State-owned Assets Supervision and Administration Commission of the State Council	SASAC
Central Committee of the Communist Youth League of China	CCYL	General Administration of Customs	CUSTOMS
National Radio and Television Administration	NRTA	Ministry of Transport	MOT
National Government Offices Administration	GGJ	Ministry of Education	MOE
National Copyright Administration	NCAC	Ministry of Science and Technology	MOST
National Development and Reform Commission	NDRC	National Forestry and Grassland Administration	FORESTRY
Cyberspace Administration of China	CAC	Civil Aviation Administration	CAAC
National Food and Strategic Reserves Administration	LSWZ	Ministry of Civil Affairs	MCA
National Energy Administration	NEA	Agricultural Development Bank	ADBC
State Administration for Market Regulation	SAMR	Ministry of Agriculture and Rural Affairs	MOA
State Taxation Administration	CHINATAX	China Meteorological Administration	CMA
China Railway	CHINA-RAILWAY	Central Patriotic Public Health Campaign Committee	CPPHCC
State Administration of Foreign Exchange	SAFE	All-China Women’s Federation	WOMEN
National Public Complains and Proposals Administration	GJXFJ	All-China Federation of Industry and Commerce	ACFIC
National Medical Products Administration	NMPA	All-China Federation of Trade Unions	ACFTU
National Healthcare Security Administration	NHSA	Ministry of Human Resources and Social Security	MOHRSS
National Immigration Administration	NIA	Ministry of Commerce	MOFCOM
State Post Bureau	SPB	National Audit Office	AUDIT
National Intellectual Property Administration	CNIPA	Ministry of Ecology and Environment	MEE
The People’s Bank of China	PBC	All-China Lawyers Association	Lawyers
China Federation of Logistics & Purchasing	CFLP	State Commission Office of Public Sectors Reform	SCOPSR
Bank of China	BOC	Ministry of Logistics	JWHQ
All-China Federation of Supply and Marketing Cooperatives	CHINACOOP	Ministry of National Defense	MOD
Committee of Political and Legal Affairs of the CPC Central Committee	CPLACPCC	Ministry of Housing and Urban-Rural Development	MOHURD
State Administration of Traditional Chinese Medicine	SATCM	Ministry of Natural Resources	MNR
Ministry of Water Resources	MWR	China Disabled Persons’ Federation	CDPF
Ministry of Justice	MOJ	China Export and Credit Insurance Corporation	SINOSURE
National Railway Administration	NRA	Red Cross Society of China	REDCROSS
National Bureau of Statistics	STATS	The Export-Import Bank of China	EXIMBANK
Ministry of Veterans Affairs	MVA	China Association for Science and Technology	CAST
Ministry of Foreign Affairs	FMPRC	Chinese Academy of Sciences	CAS
National Health Commission	NHC	China Enterprise Confederation/China Enterprise Directors Association	CEC
Ministry of Culture and Tourism	MCT	Leading Group for Rural Affairs of the CPC Central Committee	LGRACPC
State Tobacco Monopoly Administration	TOBACCO	Office of the Central Cyberspace Affairs Commission	OCCAC
China Banking and Insurance Regulatory Commission	CBIRC	Civilization Office of the Central Communist Party Committee	COCCPC
Ministry of Emergency Management	MEM	Propaganda Department of the CPC Central Committee	PDCPCC
China Securities Regulatory Commission	CSRC	The Supreme People’s Court	COURT
The United Front Work Department of CPC Central Committee	ZYTZB	The Supreme People’s Procuratorate	SPP
Organization Dept. of the Central Commission	ZYZZB		
